# Evaluating Progression Risk in Patients With Immunoglobulin A Nephropathy

**DOI:** 10.1016/j.ekir.2023.09.020

**Published:** 2023-09-22

**Authors:** Daniel C. Cattran, Jürgen Floege, Rosanna Coppo

**Affiliations:** 1 University Health Network, Toronto, Ontario, Canada; 2 Division of Nephrology and Clinical Immunology, RWTH Aachen University, Aachen, Germany; 3 Fondazione Ricerca Molinette, Regina Margherita Hospital, Turin, Italy

**Keywords:** biomarkers, children, complement, immunoglobulin A nephropathy (IgAN), prognosis, progression

## Abstract

The highly variable rate of decline in kidney function in patients with immunoglobulin A nephropathy (IgAN) provides a major clinical challenge. Predicting which patients will progress to kidney failure, and how quickly, is difficult. Multiple novel therapies are likely to be approved in the short-term, but clinicians lack the tools to identify patients most likely to benefit from specific treatments at the right time. Noninvasive and validated markers for selecting at-risk patients and longitudinal monitoring are urgently needed.

This review summarizes what is known about demographic, clinical, and histopathologic prognostic markers in the clinician’s toolkit, including the International IgAN Prediction Tool. We also briefly review what is known on these topics in children and adolescents with IgAN. Although helpful, currently used markers leave clinicians heavily reliant on histologic features from the diagnostic kidney biopsy and standard clinical data to guide treatment choice, and very few noninvasive markers reflect treatment efficacy over time. Novel prognostic and predictive markers are under clinical investigation, with considerable progress being made in markers of complement activation. Other areas of research are the interplay between gut microbiota and galactose-deficient IgA1 expression; microRNAs; imaging; artificial intelligence; and markers of fibrosis.

Given the rate of therapeutic advancement, the remaining gaps in biomarker research need to be addressed. We finish by describing our route to clinical utility of predictive and prognostic markers in IgAN. This route will provide us with the chance to improve IgAN prognosis by using robust, clinically practical markers to inform patient care.

### Evaluating Risk of Progression in IgAN: A Major Clinical Challenge

IgAN is a leading cause of chronic kidney disease (CKD). However, predicting which patients will progress to kidney failure, and how quickly, remains a major challenge for clinicians. The rate of decline in kidney function is highly variable, even in patients diagnosed in the relatively early stages of the disease. Three examples illustrate this challenge. First, the Norwegian kidney biopsy registry followed-up with 145 patients with proteinuria <1 g/d and estimated glomerular filtration rate (eGFR) >60 ml/min per 1.73 m^2^ at diagnosis for a median of 22 years. Of these, 19% of patients had a ≥50% decline in eGFR, 4 of whom had kidney failure; in contrast, 29% of patients had clinical remission (eGFR ≥60 ml/min per 1.73 m^2^, absence of hematuria and microalbuminuria, and blood pressure <140/90 mm Hg). The study found no baseline marker that could identify those at risk for progression.^1^ Second, in a Spanish study of 141 Caucasian patients who had minimal or no proteinuria at presentation, only 3.5% of patients had a >50% increase in serum creatinine after a median follow-up of 9 years (none had developed kidney failure); and in contrast, 37.5% of patients had clinical remission.^2^ Third, after a median 7-year follow-up of the VALIGA cohort (*n* = 1130), which enrolled patients from 13 European countries with median proteinuria of 1.2 g/day and mean eGFR of 74 ml/min per 1.73 m^2^, one-quarter of patients had reached the composite end point of kidney failure or a 50% decline in eGFR.^3^ The difference between these longitudinal studies in the proportion of patients who experienced disease progression or clinical remission highlights the importance of identifying those at high risk of progression (i.e., future decline in kidney function), in order to tailor treatment accordingly.

Recent advances in our understanding of IgAN pathophysiology have led to several novel agents being evaluated in patients with IgAN.^4^, ^5^, ^6^, ^7^, ^8^, ^9^ Although many of these are still in clinical trials, patients at high risk of disease progression will soon have more options. However, clinicians will need tools to match the right patient with the right treatment at the right time. Deciding whether to discontinue, continue, or intensify treatment requires longitudinal monitoring of validated markers, ideally using noninvasive measures – but which markers? Further, clinicians must identify which patients are most likely to benefit from which treatment, given potential adverse events, intensity, timing, duration, and cost – but again, using which markers?

As we will discuss, certain clinical and histological data at the time of biopsy have been widely validated for risk stratification, but their role in treatment selection has not. Further, no validated prognostic serum or urine biomarkers for IgAN other than eGFR and proteinuria exist. We are, therefore, at a crossroads, with a clear chance to improve the prognosis of this disease, but only if we have robust, clinically practical prognostic and predictive markers to inform patient care. We need to know which biomarkers and genes to assess in IgAN, in which combination, in which patients, and how often. This review examines the status quo and identifies gaps that need to be addressed to allow clinical practice to keep pace with translational research.

### Established Risk Factors Associated With Clinical Outcomes in IgAN

What do we already use to inform clinical decisions in IgAN? Uncontrolled hypertension,^10^^,^^11^ older age,^12^ obesity, and dyslipidemia^13^, ^14^, ^15^, ^16^, ^17^ are clearly associated with poor prognosis; hypertension is an especially useful prognostic marker. Smoking and nicotine have multiple deleterious effects on the kidney and are associated with the progression of kidney disease.^18^^,^^19^ These variables provide some prognostic information and allow us to make some recommendations about lifestyle management and blood pressure control. However, they do not provide direct insight into the extent of existing kidney damage or the expected rate of kidney function decline. To date, the only serum or urinary biomarkers that consistently inform on the risk of IgAN progression are baseline eGFR, which is self-explanatory, and proteinuria.^20^

Proteinuria is a marker of kidney damage, contributing to progressive CKD by promoting persistent inflammatory and fibrogenic responses.^21^ Even exposure to relatively low levels of proteinuria over time (persistent values of ≥0.5 g/d but <1 g/d) can be associated with disease progression, as demonstrated in the Validation Study of the Oxford Classification of IgAN (VALIGA) study^22^ of 1147 European patients, and in a recent analysis of the UK RaDaR IgA Nephropathy Cohort that included 923 patients with biopsy-proven IgAN and eGFR <60 ml/min per 1.73 m^2^ or proteinuria ≥0.5 g/24h.^23^ Measuring proteinuria over time is better for predicting IgAN progression compared with isolated measures at diagnosis.^24^ Sustained proteinuria (assessed as time-averaged proteinuria) or duration of proteinuria remission have significant, independent prognostic value.^25^^,^^26^ Data from 542 patients in the Toronto Glomerulonephritis Registry showed that the rate of GFR decline increased significantly with the amount of persistent proteinuria: loss of kidney function was 25 times faster in patients with sustained proteinuria >3 g/d (*n* = 121) than in those with sustained proteinuria <1 g/d (*n* = 171). Importantly, regardless of their proteinuria level at biopsy, patients who achieved even a partial remission (<1 g/d proteinuria), whether spontaneously or with intervention, progressed to kidney failure at a much slower mean rate (−0.030 ± 0.46 ml/min per 1.73 m^2^ per month) than patients who did not achieve remission (>3 g/d proteinuria; −0.719 ± 0.61 ml/min per 1.73 m^2^ per month).^25^ These observations have been widely supported. In an individual participant meta-analysis of 1037 patients from 12 randomized controlled trials, early change in proteinuria was found to predict eGFR slope in IgAN.^27^ This supported the use of proteinuria reduction as a surrogate end point for loss of kidney function and progression to kidney failure in new IgAN clinical trials.^27^^,^^28^ Such is the strength of evidence linking proteinuria over time with IgAN progression that complete remission of proteinuria is now a clear goal of clinical care.

Other measures obtained from urinalysis have the potential to be informative about IgAN progression, though the relationship has yet to be defined. In particular, the role of hematuria in IgAN prognosis is not clear-cut, partly because there is no internationally validated, straightforward, or standardized measure; and partly because the requirement for fresh urine samples makes it difficult to measure centrally in global clinical trials. Automated laboratory methods may eventually allow us to standardize the quantification of microscopic hematuria, although there are a number of analytical challenges to overcome.^29^ Asymptomatic hematuria is common among patients presenting with IgAN, but one recent meta-analysis (*n* = 5660) showed that initial microscopic hematuria, including mild hematuria (1–29 red blood cells/high-power field, or 1+/2+ by dipstick), was associated with a significant 87% increase in the risk of kidney failure in the long-term. Conversely, macroscopic hematuria was associated with a significant 32% decrease in risk^30^; however, this could be interpreted as lead-time bias because macroscopic hematuria occurs most frequently in children and young adults, or it may trigger an adult patient to seek earlier clinical attention. Although less well studied so far, persistent hematuria may have relevant prognostic value. In a retrospective study (*n* =1333), time-averaged hematuria during follow-up was a significant, independent predictor for kidney failure or a 50% decline in eGFR.^31^ Other longitudinal studies have also reported the association between time-averaged hematuria, or hematuria remission, with disease progression in IgAN.^30^^,^^32^, ^33^, ^34^, ^35^ For example, in a cohort of 112 patients with IgAN, after a mean follow-up of 14 years, time-averaged hematuria was found (among other markers) to be an independent predictor of kidney failure in a multivariate analysis. After hematuria disappeared, the rate of decline in kidney function slowed significantly.^32^

Because IgAN requires a biopsy for diagnosis, histopathology of the kidney provides crucial prognostic information to guide IgAN management, typically via the widely used Oxford MEST (mesangial [M] and endocapillary [E] hypercellularity, segmental glomerulosclerosis [S], interstitial fibrosis/tubular atrophy [T]) score. Analysis of 3 large observational data sets showed that the lesions included in the original Oxford MEST score have a predictive value that is independent of clinical factors.^36^ More recently, crescent formation has been added as a graded parameter in a modified version of the MEST classification (MEST-C),^37^^,^^38^ because it is independently associated with the deterioration of kidney function in patients who are not receiving immunosuppressive treatment. Even in patients who have received corticosteroids and/or other immunosuppressive agents, cellular or fibrocellular crescents in ≥25% of glomeruli is still independently associated with a ≥50% reduction in GFR or kidney failure.^37^^,^^39^, ^40^, ^41^ Of the MEST components, the T score consistently demonstrated the highest predictive value in a meta-analysis.^42^ However, the combination of all MEST lesions into one score does add clinical value: in the long-term follow-up of the VALIGA cohort of European patients with IgAN, the M1, S1, and T1–T2 lesions, as well as the whole MEST score were independently related with the combined end point of 50% decline in eGFR or kidney failure. There was no effect modification by age for these associations, suggesting that they are valid in both adults and children. C lesions were independently associated with the rate of decline in kidney function in patients who had not been treated with immunosuppression; however, this association was not detectable in treated cases.^3^

MEST-C may also have some value in treatment selection. In a retrospective, propensity score matching study in 858 patients from Japan (one-third of whom received renin-angiotensin system blockers), patients with M1, E1, S1, C1 + 2, and T0 scores were likely to benefit from corticosteroids. In contrast, patients with substantial tubulointerstitial scarring (T1 + 2) scores were less likely to respond.^43^ This finding has been validated in 184 matched patients from the VALIGA cohort.^44^

In summary, until recently, clinicians relied on broad categorizations based on a small number of markers to estimate the extremely heterogeneous rate of kidney function decline in patients with IgAN. Could the integration of these clinical and histopathologic markers improve prognostic accuracy?

### Combining Risk Factors to Increase Accuracy: The International IgAN Prediction Tool

The International IgAN Prediction Tool combines clinical, histopathologic, and demographic variables, as well as the use of renin-angiotensin system blockers and immunosuppression at biopsy. Based on the model, patients are assigned to risk categories, which show each patient’s mean predicted risk of experiencing either a 50% decline in eGFR or progression to kidney failure within up to 80 months from biopsy.^45^^,^^46^ The tool was validated in an autonomous, large group of predominantly White, Chinese, and Japanese patients with a broad spectrum of disease severity,^45^ and subsequently in smaller external cohorts of Chinese and White patients.^47^^,^^48^ It comprises 2 models – one which includes ethnicity and one which does not–because it has not been validated in all ethnic groups.^45^^,^^49^ Both models identify patients with aggressive IgAN: an increase in predicted risk of progression is associated with a faster rate of eGFR decline. Per the 2021 Kidney Disease: Improving Global Outcomes guidelines, the IgAN Prediction Tool is currently the preferred method of evaluating the risk of progression.^20^

The International IgAN Prediction Tool has been validated in a large external cohort of Chinese and Argentinian patients, 73% of whom were on renin-angiotensin system blockers treatment before biopsy, to ensure that it can be retained as clinical practice evolves; this may be more representative of current clinical practice than the original validation cohort, in which that proportion was 30%.^50^ Further study has demonstrated that after modification of the parameters that are entered, the tool can be used to assess prognosis 1–2 years after biopsy; the majority (71%) of this test population had received renin-angiotensin system blockers 1 year after diagnosis, and 41% had received immunosuppressive treatment between a diagnostic biopsy and follow-up.^51^ Validation for use up to 2 years after biopsy is an important extension because the tool can now be applied at clinically relevant timepoints post-biopsy and may identify a subgroup of patients at low risk of progression, in whom the frequency of monitoring can be reduced safely. However, when used this way, the tool’s prognostic value has only been validated for the next 4 years,^51^ which is a relatively short time in the overall context of IgAN progression. Simulation of risk-based treatment allocation indicates that the use of the International IgAN Prediction Tool does improve treatment choices compared with using proteinuria levels alone: more high-risk patients were allocated to receive immunosuppression, and fewer patients who were low-risk received unnecessary treatment.^52^

Despite its contribution to improving clinical care in IgAN, the International IgAN Prediction Tool only has prognostic value–current Kidney Disease: Improving Global Outcomes guidelines state that the tool should not be used to assess the likelihood of treatment response in an individual patient.^20^ Another limitation, already discussed above, is that validation has so far been based on retrospective cohorts, some of which did not receive the same level of supportive care as we would recommend today; prospective validation of the tool is needed. In addition, here has been no validation of the tool in patients receiving treatment with sodium-glucose cotransporter-2 inhibitors or any other emerging therapies (e.g., endothelin-A receptor blockers, targeted release formulation of budesonide [Nefecon], complement inhibitors, etc.), which are increasingly relevant cohorts within the IgAN patient population. Further, the online calculator excludes patients with an eGFR <15 ml/min per 1.73 m^2^,^46^ which may prevent identification of patients who have acute kidney injury.^53^ Finally, validation of the tool would also be welcome in patients from different geographies and ethnicities, because the majority of patients in the cohorts used for derivation and validation of the tool were described as either White, Japanese, or Chinese.^49^^,^^51^

Therefore, the status quo is that clinicians treating patients with IgAN rely heavily on data obtained at biopsy, with very few biomarkers to guide the choice of therapy or reflect the efficacy of therapy without doing another biopsy. It is difficult to ascertain the extent of fibrosis and measure structural changes in the kidney over time. Further, because novel therapies are introduced into the treatment algorithm, predictive markers to aid patient selection will be increasingly important. In the next sections, we consider potential novel biomarkers in IgAN (Figure 1) and some critical limitations in the existing evidence base.

**Figure 1:**
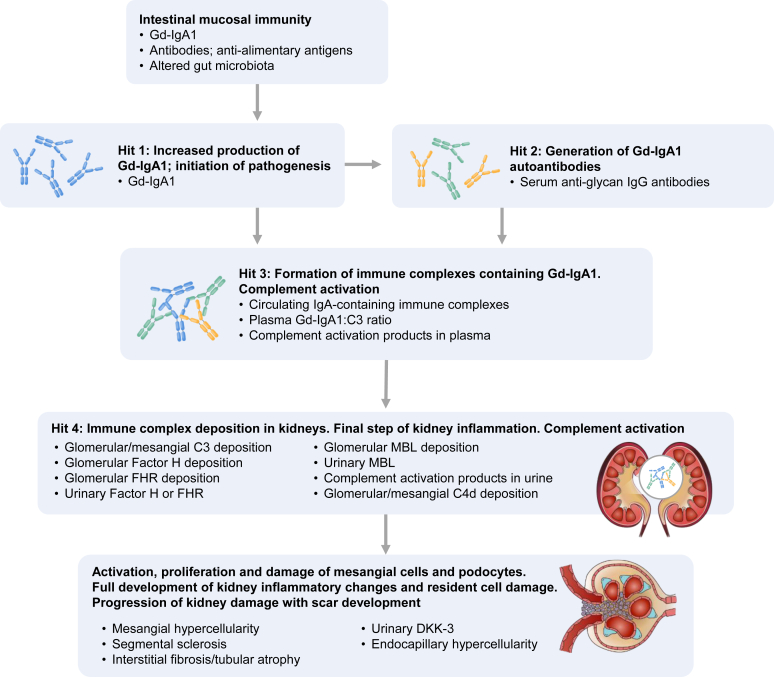
Selected potential novel markers of IgAN progression by stage of disease pathogenesis. C3, complement 3; CD, cluster of differentiation; DKK-3, urinary Dickkopf-3; FHR, Factor H-related proteins; GD-IgA1, galactose-deficient IgA1; IgAN, immunoglobulin A nephropathy; MBL, mannose-binding lectin.

### Looking Ahead: An Update on Markers of Progression

#### Markers of Complement Activation

Owing to the role of complement in IgAN pathogenesis,^54^^,^^55^ quantification of complement activity in kidney tissue, plasma, and urine is likely to have implications for prognosis and choice of treatment. Complement activation in IgAN can already be observed at diagnosis: mesangial codeposition of C3, a product of the complement alternative pathway, is present in >70% of kidney biopsies^56^ that are classically dominated by mesangial deposits of IgA over the other classes of immunoglobulin.^57^ Properdin, C3dg, C5b-9, Factor B, Factor H, and Factor H-related proteins (FHRs), all markers of the alternative and terminal pathways of complement activation, have also been identified in diagnostic biopsies, as well as markers of lectin pathway activation: mannose-binding lectin, C4d, L-ficolin, MASP2, and MASP1/3.^56^^,^^58^^,^^59^

Biopsy analyses have linked complement activation, identified by glomerular C3 deposition, with glomerular injury, inflammation, and a high risk of disease progression.^58^ For example, in a study of 821 patients with IgAN, those with glomerular C3 deposition at biopsy had a significantly higher MEST-C score and were more likely to have crescents and interstitial inflammatory cell infiltration than those classified as C3-negative.^60^ In another study of 453 patients with IgAN, component lesions of the MEST-C score, namely M1, S1, T1–2, and crescentic lesion, were significantly correlated with mesangial C3 deposition, defined as an immunofluorescence intensity of C3 ≥2+ within the mesangium. In this study, combining the C3 deposition measure with the individual lesions represented in the MEST-C score improved the predictive accuracy for IgAN progression.^61^

Complement activation has also been associated with crescent formation in IgAN by measuring the urinary concentration of mannose-binding lectin, Bb, C4d, C3a, C5a, and soluble C5b-9 in a study of 100 patients.^62^ A cautionary note in the interpretation of these findings is that urinary complement levels might be influenced by proteinuria, including spillage of complement factors into the urine from serum. Serum markers of complement activation have also been investigated: low circulating levels of C3 (which likely mirror more intense C3 deposition) have been linked with a decline in kidney function.^63^^,^^64^ Serum levels of circulating IgA-containing immune complexes have also been associated with disease activity.^65^ Published data on the prognostic value of plasma galactose-deficient IgA1 (Gd-IgA1) have provided inconsistent results.^66^, ^67^, ^68^, ^69^, ^70^ However, considering Gd-IgA1 in tandem with C3 concentration may improve its prognostic value: plasma Gd-IgA1:C3 ratio has been reported as an independent marker of IgAN progression, at least in Asian cohorts.^71^, ^72^, ^73^ The Gd-IgA1:C3 ratio indicates the extent of complement activation, which contributes to kidney injury in IgAN. The discrepancies observed between published reports on plasma Gd-IgA1 could be explained by using different lectins in assays, which may not provide comparable results.^66^ Notably, the association of IgG antiglycan antibodies increases the nephrotoxicity of Gd-IgA1 via complement activation.^74^

Specific markers of the AP, Factor H, and FHRs are likely markers of disease progression in IgAN, but their roles are less clear-cut. Multiple studies have shown that elevated levels of urinary Factor H (or possibly FHRs, given their homology) are associated with features of IgAN progression, including interstitial fibrosis, glomerular sclerosis, tubulointerstitial damage, and an overall decline in kidney function.^75^, ^76^, ^77^ However, it is possible that these observations actually reflect proteinuria; more robust controls are needed. Nonetheless, data from biopsies in a UK cohort study and a Norwegian proteomics study support this finding concerning FHRs, showing that abundant glomerular FHR5 is associated with IgAN progression.^78^^,^^79^ The data are less conclusive with regard to Factor H: whereas glomerular Factor H was significantly more abundant in patients with progressive disease in the proteomics study, the opposite pattern was seen in the cohort study, with Factor H significantly reduced in patients with progressive disease compared with stable disease.^78^

Turning to markers of lectin pathway activation, these have been linked to disease activity and progression in about one-quarter of patients with IgAN.^80^ Glomerular deposition of mannose-binding lectin has been significantly associated with increased mesangial and extracapillary proliferation, interstitial infiltration, sclerosis, and increased proteinuria.^80^ Urinary mannose-binding lectin has also been associated with disease severity, indicated by hypertension, eGFR, and magnitude of proteinuria.^81^ Multiple studies have demonstrated that glomerular or mesangial deposition of C4d (and absence of C1q) has independent prognostic value in IgAN.^59^^,^^82^, ^83^, ^84^, ^85^ Interestingly, although fewer data have been published so far, arteriolar deposition of C4d may have a certain prognostic value.^86^

Staining of biopsy samples using assays for different components of the complement pathways should ideally be standardized and widely used; it represents a key area where clinically valuable insights can already be obtained. Although it is true that alternative methods may be more practical, it may be more difficult to remove confounding factors. For example, urinary excretion of C4d or the C4d-to-creatinine ratio was an independent predictor of progression to kidney failure in one cohort study of 168 patients with IgAN and crescent lesions.^87^ However, this marker may be more likely to reflect current disease activity rather than provide prognostic information.

#### Tackling Gd-IgA1 Production From Another Angle: The Link to Gut Microbiota

IgAN pathogenesis is associated with impaired function of the intestinal mucosal barrier and potentially with an imbalance in the associated bacterial populations; in predisposed patients, this may favor an inflammatory response and increase Gd-IgA1 production, as seen in Gd-IgA1 levels in serum and urine samples from patients with IgAN.^88^ It is therefore possible that gut microbiota may provide predictive or prognostic markers in the future.^88^ For example, one prospective study (*n* = 127, with 86 matched healthy controls) linked changes in specific gut microbe populations, notably expansion of *Escherichia-Shigella*, with IgAN; this change was reversed in patients who responded to immunosuppression.^89^ Another study compared tonsil and stool microbiome profiles in a cohort of 93 patients with IgAN (median proteinuria: 1.5 g/d) to 58 household-matched, unrelated control participants. Those with IgAN had an expanded population of *Neisseria* in their tonsils and increased serum production of anti-*Neisseria* IgA. However, no differences in stool microbiota were observed between the 2 groups.^90^ This area warrants further study, especially given the development of novel therapies designed to attenuate Gd-IgA1 production, such as the gut-directed formulation of budesonide.^5^ A note of caution here: extremely precise, well-controlled study designs will be needed to establish robust data sets in this area, given the variation in microbiomes and diet between individuals with CKD or other kidney diseases and in patients of different ethnicities.

#### MicroRNAs

MicroRNAs may help determine the risk of progression in IgAN; some of these noncoding RNAs have a role in controlling immune response and kidney fibrosis. One small pilot study investigated 4 microRNAs that were differentially expressed in kidney biopsies from IgAN progressors versus non-progressors. Of the 4, all increased the discrimination score of the International IgAN Prediction Tool, although none reached significance; miR-150-5p was the most strongly associated with risk of progression.^91^ In a different study, incorporating miR-204 expression into the International IgAN Prediction Tool significantly increased its accuracy in predicting disease progression.^92^

#### Other Potential Markers of IgAN Progression

Various other potential markers of IgAN progression have been proposed, but none have been validated. Few studies include appropriate comparator groups, for example, controls with non-IgAN glomerular diseases; or adjust for nonspecific effects of CKD stage. Potential markers include interstitial kidney inflammation,^93^ anemia,^94^ hyperuricemia, and elevated serum uric acid.^95^ Increased plasma osmolarity,^96^ urinary MMP-7,^97^ and elevated neutrophil to lymphocyte ratio^98^^,^^99^ have also been considered as possible markers of progressive decline in kidney function.

Finally, the use of artificial intelligence in kidney pathology has shown promising accuracy in identifying patients with IgAN at high risk of disease progression. Automated prediction of kidney failure using a deep learning predictive score based on 496 biopsy images from 442 patients was shown to be noninferior to MEST-C in terms of prognostic accuracy in a randomly selected subgroup of 95 samples. Although the deep learning predictive score correlated significantly with the tubulointerstitial score, there was no correlation with mesangial, endocapillary, segmental sclerosis, and crescent parameters. However, the artificial intelligence approach incorporated interstitial fibrosis and hyaline casts within the deep learning predictive score. These parameters reflect reduced GFR (interstitial fibrosis) and proteinuria (hyaline casts), calling into question the added clinical value of deep learning predictive score over MEST-C so far.^100^ In a different approach, neural networks have been trained to predict the development of kidney failure in patients with IgAN. One of these showed ≥90% accuracy in predicting kidney failure status in retrospective test cohorts, including 1040 patients overall,^101^ and the other showed 86% prognostic accuracy (Harrell C index) out to 10 years (*n* = 948).^102^ Other novel methods can provide proteomics and transcriptomics with spatial distribution, which could complement classical histopathology data.^103^

### Longitudinal Assessment of Kidney Damage

Although it is difficult to monitor kidney fibrosis noninvasively, imaging techniques such as diffusion kurtosis imaging may have clinical value in the long term.^104^ Another option for quantifying changes in molecular drivers of disease over time is to use molecular imaging such as ^18^F-FDG-PET to identify inflammation or other probes to assess fibrosis.^105^ These methods need to be evaluated in large studies before they can be translated confidently into clinical practice.

In the meantime, a study including 49 patients with IgAN showed that serum endotrophin, a molecule present in fibrotic kidneys, was associated with interstitial fibrosis in biopsies in a multiple regression analysis.^106^ This holds promise for noninterventional methods of assessing kidney fibrosis. Another marker of fibrosis is urinary Dickkopf-3, a glycoprotein associated with tubular damage shown to predict short-term eGFR loss.^107^ High urinary Dickkopf-3 concentrations appear to predict the short-term progression of CKD of various etiologies, serving as a marker of subclinical kidney injury.^107^^,^^108^ It has been reported that urinary Dickkopf-3 normalizes within 30 days of treatment initiation in patients with C3 glomerulonephritis, granulomatosis with polyangiitis, and microscopic polyangiitis, highlighting its additional potential for monitoring treatment response; further study of this question is required.^108^^,^^109^

### Additional Markers of Patient Selection and Treatment Response

Many of the prognostic markers discussed above are likely to also predict response to certain treatments, although evidence for this from prospective trials is lacking. A handful of additional markers have been solely and specifically evaluated as markers of patient selection and likelihood of response to treatment. For example, a prospective study evaluated whether the intensity of inflammatory cell infiltration in the kidney could predict response to immunotherapy in 621 Chinese patients with IgAN at high risk of progression. Analysis of cellular infiltrates in biopsy specimens showed that patients with samples in the highest tertile of CD206+ macrophage infiltration in the glomeruli were 40 times more likely to respond to immunosuppression compared with those in the lowest tertile.^110^ A similar pattern was seen for infiltration of CD68+ macrophages in the glomerular area.^110^

Another example of a biomarker that may be useful for patient selection is Gd-IgA1, the first “hit” in IgAN pathogenesis. A high concentration of circulating Gd-IgA1 is a feature of IgAN but insufficient to account for the full spectrum of kidney damage associated with the disease. However, it may be informative in patients for whom a transplant is an option: a subset of patients who presented with clinically severe IgAN, with higher serum levels of Gd-IgA1 than others, appeared to be more likely to experience disease recurrence after transplant.^111^ A meta-analysis (*N* = 515) supports this, indicating that posttransplant (but not pretransplant) serum Gd-IgA1 level is associated with IgAN recurrence.^112^ Levels of corresponding autoantibodies, predominantly IgG autoantibodies, have been found to correlate with serum Gd-IgA1 levels in patients with IgAN,^113^ and have been shown in mice to have a pathogenic role.^74^ These observations make these an additional possible marker to indicate IgAN recurrence posttransplant.^114^ However, further studies are needed to precisely determine the pattern of correlation and the optimal timepoint at which these markers should be assessed in relation to transplant. There is also a clear need for practical and affordable assays to support clinical implementation once the evidence base is established.

### Markers of IgAN Progression in Children

The trajectory of eGFR over time differs between children and adults with IgAN. In a multiethnic cohort of 1060 children with IgAN, the trajectory was typically nonlinear, first showing an initial increase in eGFR after kidney biopsy, followed by a plateau, before a linear decline a few years later similar to that seen in adults.^115^ These children had frequently received corticosteroid treatment (14% before and in 58% after kidney biopsy).^115^ It appears that children with IgAN have a greater kidney recovery reserve or compensatory functionality (e.g., hyperfiltration) than adults, producing a slower rate of overall eGFR decline.^116^ However, many children exhibit proteinuria as adults even if they did not reach clinical end points during childhood, or sometimes even if they had proteinuria remission or minimal proteinuria with microscopic hematuria during childhood.^116^ Despite the early course of IgAN appearing to be clinically different in adults and children, it is now clear that disease progression does occur in children, making risk assessment equally important, with similar challenges to those in adults.

Multivariate analysis of the VALIGA data set in patients <18 and <23 years of age showed that eGFR and proteinuria at biopsy did not predict progression. However, similar to adults, time-averaged proteinuria had significant predictive value, as did mean arterial blood pressure at biopsy and over time.^117^

Validation of the MEST score has been difficult in children, with studies often underpowered because they rely on the attainment of end points such as a 50% drop in eGFR from the rate at biopsy or kidney failure. These are unusual events detected in only 5% of 1060 cases included in an evaluation of the International IgAN Prediction Tool in children (<18 years of age) from various continents.^115^ However, as in adults, T lesions appear to be the strongest of the MEST-C risk factors for progression in children.^118^ Combining clinical features, such as proteinuria, with MEST-C scores may be a more informative approach to risk assessment in children.^115^ The updated International IgAN Prediction Tool for use in children reflects the nonlinear progression pattern and may permit more precise analyses.^115^

Early research indicates that some novel markers identified in adult patients with IgAN may also have clinical value in children. In one study in 44 children with IgAN, high serum IgA:C3 ratio together with extensive glomerular C3 staining were associated with persistent kidney inflammation, potentially predicting IgAN progression.^119^ In another study, in 98 children with IgAN, mesangial C3 deposition and serum C3 concentration were associated with kidney outcome.^64^

### Remaining Gaps in Biomarker Research: What is Our Route to Clinical Utility?

Many of the data sets described above are small, single-center, retrospective studies that sometimes provide conflicting evidence. Although these are valuable starting points for large, prospective studies, their interpretation for everyday clinical practice is limited. We propose some points for future biomarker research to help clear the path to clinical implementation (Figure 2) as follows:

1. Given the highly variable incidence and severity of disease across geographies and ethnicities, validation studies should ideally be done in multiethnic populations. Studies performed exclusively in one location or ethnic population should be replicated in others.
2. Although potentially complicated, an essential part of validation studies will also be to account for the impact of additional immunologic diseases or other comorbidities on kidney function. Without controlling for this, it will be difficult to robustly imply the causation of a decline in kidney function and identify clinically reliable biomarkers.
3. The search for new biomarkers also needs to consider the systematic nonspecific effects of kidney function. Examples of these potential confounding effects include retention of low molecular weight proteins in serum at low GFR; low molecular weight proteinuria, typical of widespread tubular damage; and glomerular spillage of larger serum proteins that may simply reflect proteinuria.
4. Development of validated, practical assays that can be used in the clinical setting, not only research-grade assays, is needed. A standardized methodology will be needed to address problems such as discrepancies in the assays used or in urinalysis that may be due to differences in expertise between clinical sites.
5. As novel risk factors are validated, they need to be integrated into the International IgAN Prediction Tool (and demonstrate added prognostic value) or be included in the creation of a different risk prediction tool. If this is not possible, guidance on which measures take precedence over others will be needed.
6. The confounding effect of genetic factors, such as collagen IV mutations associated with IgAN, has to be considered and further investigated.
7. Over time, biomarker research will need to turn to questions associated with the long-term treatment of patients with IgAN, for example in patients who have already received immunosuppressive treatment and shown a marked reduction of proteinuria but later show a relapse in proteinuria.

**Figure 2:**
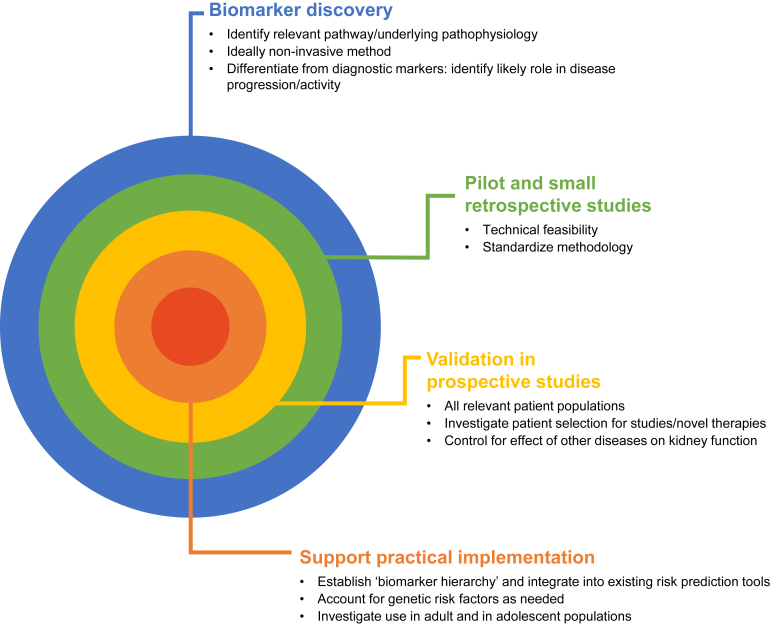
Pathway to the clinical utility of novel biomarkers in IgAN. IgAN, immunoglobulin A nephropathy.

### Conclusion

#### A Call to Action to Give Biomarker Research the Focus Our Patients Deserve

At present, there is no robust basis for changing the current Kidney Disease: Improving Global Outcomes guidance that there are no accepted serum or urine biomarkers for IgAN progression apart from eGFR and proteinuria. However, the importance of establishing a prognosis and predicting treatment efficacy cannot be overstated for patients with IgAN and their caregivers. Establishing a prognosis can alleviate anxiety if the risk of progression is low or provide a rationale for more intensive management if the risk of progression is high. Noninvasive monitoring of specific risk factors and determining their independent significance can also guide treatment selection and sequence. This is increasingly important now that novel drugs are in the latter stages of clinical development^7^^,^^120^, ^121^, ^122^, ^123^, ^124^ or recently approved, such as the targeted release formulation of budesonide (Nefecon) and sparsentan.

In addition to considerations of everyday clinical decision-making, incorporating risk stratification into clinical trials will improve the power of studies by ensuring that patients’ risk profiles are matched with an appropriate trial. It is hoped that ongoing registries, such as the Canadian Glomerulonephritis Registry–linked to the Translational Research Initiative – and the National Kidney Foundation Patient Network, will provide further insights into IgAN progression and corresponding markers.^125^^,^^126^ Eventually, a meticulous, tailored management approach may delay or prevent progression to dialysis and increase rates of complete and permanent remission in patients with IgAN.

## Disclosure

DCC has received payment for expert testimony from the Canadian Medical Protection Association; has participated in a Data Safety Monitoring Board or Advisory Board for Alexion, Alnylam, Calliditis, ChemoCentryx, Dimerix, Kyowa Hakko Kirin Co, Novartis, Reistone, Vera Therapeutics, and Zyversa; and has a leadership or fiduciary role in other board, society, committee, or advocacy group, paid or unpaid, for NephCure, NephSAP/ASN, SONG-GN, and UpToDate. RC has worked as Scientific Advisor for Alnylam, ARGENX, Calliditas, Chinook, Menarini, Novartis, Ostuka-Visterra, Purespring, Reata, STADApharm, and Travere; serves on the Data Safety Monitoring Board in clinical trials of AMGEN and Bayer; and receives honoraria as Section Editor of UpToDate. JF has received consultancy fees and/or honoraria from AstraZeneca, Calliditas, Chinook, Novartis, Omeros, STADApharm, and Travere; and serves on the Data Safety Monitoring Board in clinical trials of NovoNordisk and Visterra.
